# Supplementary material for: Getting More Out of Biomedical Documents with GATE's Full Lifecycle Open Source Text Analytics
Source: PLoS Comput Biol. 2013 Feb 7;9(2):e1002854. doi: 10.1371/journal.pcbi.1002854 (PMC3567135; doi:10.1371/journal.pcbi.1002854)
Supplement: Dataset S1 — GATE software. Dataset S1 bundled with this paper contains a distribution of GATE (or see http://gate.ac.uk/download/). (TGZ) [file pcbi.1002854.s001.tgz › plos-gate/plugins/Tagger_MetaMap/doc/index-files/index-12.html]

V-Index


---


|  |  |  |
| --- | --- | --- |
| **Overview** \| Package \| Class \| Use \| **Tree** \| **Deprecated** \| **Index** \| **Help** | |  |
| **PREV LETTER**   NEXT LETTER | **FRAMES**    **NO FRAMES**     **All Classes** |  |


D E F G I M N O P S T V 

---


## **V**

**valueOf(String)** - Static method in enum gate.metamap.OutputMode: Returns the enum constant of this type with the specified name. **valueOf(String)** - Static method in enum gate.metamap.TaggerMode: Returns the enum constant of this type with the specified name. **values()** - Static method in enum gate.metamap.OutputMode: Returns an array containing the constants of this enum type, in the order they are declared. **values()** - Static method in enum gate.metamap.TaggerMode: Returns an array containing the constants of this enum type, in the order they are declared. **VOID** - Static variable in interface info.olteanu.interfaces.StringFilter

---


|  |  |  |
| --- | --- | --- |
| **Overview** \| Package \| Class \| Use \| **Tree** \| **Deprecated** \| **Index** \| **Help** | |  |
| **PREV LETTER**   NEXT LETTER | **FRAMES**    **NO FRAMES**     **All Classes** |  |


D E F G I M N O P S T V 

---
